# Effects of external cephalic version for breech presentation at or near term in high-resource settings: A systematic review of randomized and non-randomized studies

**DOI:** 10.18332/ejm/128364

**Published:** 2020-11-20

**Authors:** Aase S. Devold Pay, Katarina Johansen, Anne C. Staff, Katariina H. Laine, Ellen Blix, Inger Økland

**Affiliations:** 1 Department of Gynecology and Obstetrics, Division of Women Health, Oslo University Hospital, Oslo, Norway; 2 Department of Nursing and Health Promotion, Faculty of Health Sciences, Oslo Metropolitan University, Oslo, Norway; 3 Institute of Clinical Medicine, University of Oslo, Oslo, Norway; 4 Department of Obstetrics and Gynecology, Stavanger University Hospital, Stavanger, Norway; 5 Department of Caring and Ethics, Faculty of Health Sciences, University of Stavanger, Stavanger, Norway

**Keywords:** systematic review, mode of delivery, breech birth, external cephalic version

## Abstract

**INTRODUCTION:**

External cephalic version (ECV) for breech presentation involves manual manipulation of the fetus from breech to cephalic presentation at or near term, in an attempt to avoid breech birth. This systematic review summarizes the literature on the effects of ECV at or near term on pregnancy outcomes in high-resource settings.

**METHODS:**

The MEDLINE, Embase, CINAHL, Cochrane Library, MIDIRS, and SweMED+ databases were searched for relevant articles published through April 2019, with no limitation on publication date. Clinical trials comparing the effects of ECV at ≥36 weeks, with or without tocolysis, with that of no ECV, conducted in northern, western, and central Europe, the USA, Canada, Australia, and New Zealand were eligible for inclusion.

**RESULTS:**

Nine articles reporting on 184704 breech pregnancies were included. Pooled data showed that ECV attempts reduced the failure to achieve vaginal cephalic birth (risk ratio, RR=0.56; 95% CI: 0.45–0.71), caesarean section performance (RR=0.57; 95% CI: 0.50–0.64), and non-cephalic presentation at birth (RR=0.45; 95% CI: 0.29–0.68) compared with no ECV. ECV attempts also increased the incidence of Apgar score <7 at 5 minutes (RR=1.29; 95% CI: 1.10–1.52).

**CONCLUSIONS:**

Women for whom ECV is attempted at or near term are at reduced risk of caesarean section, non-cephalic presentation at term, and failure to achieve vaginal cephalic birth. Compared with no ECV, attempted ECV was also associated with a slightly increased risk of a low Apgar score at 5 minutes. The evidence is limited by the scarcity of high-quality research and the presence of risks of bias.

## INTRODUCTION

Breech presentation, which occurs in approximately 3–4% of fetuses at term, is defined as the longitudinal positioning of a fetus with the buttocks or feet closest to the cervix. Multiple factors, including placenta previa, maternal hypothyroidism, multiple gestations, uterine and pelvic abnormalities, and fetal factors such as anencephaly, neurological impairment, and prematurity, may cause a fetus to present in breech^1,2^. However, no etiological explanation can be offered in approximately three-fourths of all term breech presentations^3^. Even in the absence of an underlying fetal or maternal abnormality, breech presentation increases the risks of delivery complications and adverse fetal outcomes^4^.

External cephalic version (ECV) involves the application of targeted manual pressure on the maternal abdominal wall to manipulate the fetus from breech presentation into a cephalic position. It is performed as an elective procedure in non-laboring women at or near term to improve the chance of vaginal cephalic birth. A meta-analysis showed that the success rate of ECV ranges from 16% to 100%, with a pooled rate of 58%^5^. Predictive variables for successful ECV include parity, placental location, breech engagement, palpability of the fetal head, and practitioners experience^6^.

The effectiveness of ECV is based on its ability to increase the proportion of fetuses in cephalic presentation at birth, and thereby decrease the frequency of cesarean section (CS). This effectiveness is supported by a systematic review of eight randomized controlled trials (RCTs) of ECV at term (1308 women)^7^; compared with no ECV attempt for breech fetuses, attempted ECV reduced the risks of non-cephalic presentation at birth and CS by approximately 60% and 40%, respectively. These findings and other supportive evidence informed the Cochrane Foundation’s recommendation that ECV be offered to non-laboring pregnant women with uncomplicated singleton breech presentation at ≥36 weeks^7^. In addition, the American and Royal Colleges of Obstetricians and Gynecologists recommend the use of ECV as first-line management of breech presentation at term^8,9^.

The applicability of the Cochrane recommendation in Nordic countries is unclear because maternal and fetal factors differ between these countries and the lowerresource countries from which most of the evidence supporting this recommendation was derived. Although ECV has been found to decrease the frequency of CS compared with no ECV, and international guidelines recommend that women be advised to choose attempted ECV, no universal recommendation on ECV has been established for Nordic countries.

### Objectives

The purpose of this systematic review was to summarize research on the effects of ECV at or near term on measures of pregnancy outcomes in high-resource settings.

The protocol for this review has been registered in the PROSPERO international prospective register of systematic reviews (no. CRD42017062455).

## METHODS

### Literature search strategy

Electronic databases (Medline, Embase, CINAHL, Cochrane Library, MIDIRS, and SweMED+) were searched to identify eligible studies from the earliest year possible through April 2019, published in English or a Scandinavian language. The search strategy was developed for Medline and modified for use in other databases. The Medline search string was: [Breech Presentation/] OR [breech.tw,kf.] OR [Version, Fetal/] OR [(external adj2 version*).tw,kf.] OR [cephalic version*.tw,kf.]. The reference lists of relevant studies were searched manually to identify additional studies. We conducted the search and reported the findings according to the Preferred Reporting Items for Systematic Reviews and Meta-Analyses guidelines (PRISMA Checklist given in Supplementary file)^10^.

### Study selection and data extraction

Studies examining pregnant women with singleton fetuses in breech presentation at or near term (≥36 weeks) and no ECV contraindication, conducted in the US, Canada, Australia, New Zealand, and the northern, western, and central European countries categorized as ‘very high’ on the United Nations’ Human Development Index^11^ (to maximize applicability of the findings to women in Nordic countries) were included. Eligible studies compared the intervention of ECV near or at term (with or without tocolysis) with no ECV, with the primary outcomes of the failure to achieve vaginal cephalic birth (CS + breech presentation) and CS and the secondary outcomes of non-cephalic presentation at delivery, vaginal breech birth, Apgar scores <7 at 1 minute and at 5 minutes, perinatal death, and neonatal intensive care units (NICU) admission. Observational (cohort and nested case-control) studies, RCTs, intervention studies, and systematic reviews were eligible for inclusion in the review.

A list of articles meeting the inclusion criteria based on titles and abstracts was compiled. The full texts of these studies and those of uncertain relevance were retrieved. Two authors independently evaluated the studies’ fulfillment of the inclusion criteria, with any discrepancy discussed with a third until a final set of relevant studies was agreed upon. The following data were extracted from all included studies: general information (authors, publication year, country of investigation), population (number of participants), intervention, comparison group (number of participants), study design, and outcomes of interest.

### Assessment and synthesis

Two authors assessed the risk of bias for each study. The assessment of RCT quality and risk of bias (selection, performance, detection, attrition, reporting, and other) was conducted using the Cochrane Collaboration’s risk-of-bias tool for RCTs^12^. The risk-of-bias tool in non-randomized studies^13^ was used to assess the quality of non-randomized studies (NRSs), which included the examination of confounding, co-interventions, selection bias, deviations from intended interventions, missing data, outcome measurement, and reporting of results.

A meta-analysis was conducted to pool risk ratios (RRs) for the study outcomes. The degree of statistical heterogeneity was assessed using the inconsistency index (I²). A random effect model was used when I²>50%, and a fixed effect model was used otherwise. The results are reported with 95% confidence intervals (CIs), and p values <0.05 were considered to be significant. All computations were performed using the RevMan software (version 5.3; The Nordic Cochrane Center, The Cochrane Collaboration).

We did not perform a sensitivity analysis to assess the effects of the risks of bias of the studies included in the main effects analysis, as all seven NRSs^14-20^ were at serious risk of bias (Table 3). The two RCTs^21,22^ had unclear risk of bias.

## RESULTS

**Figure 1:**
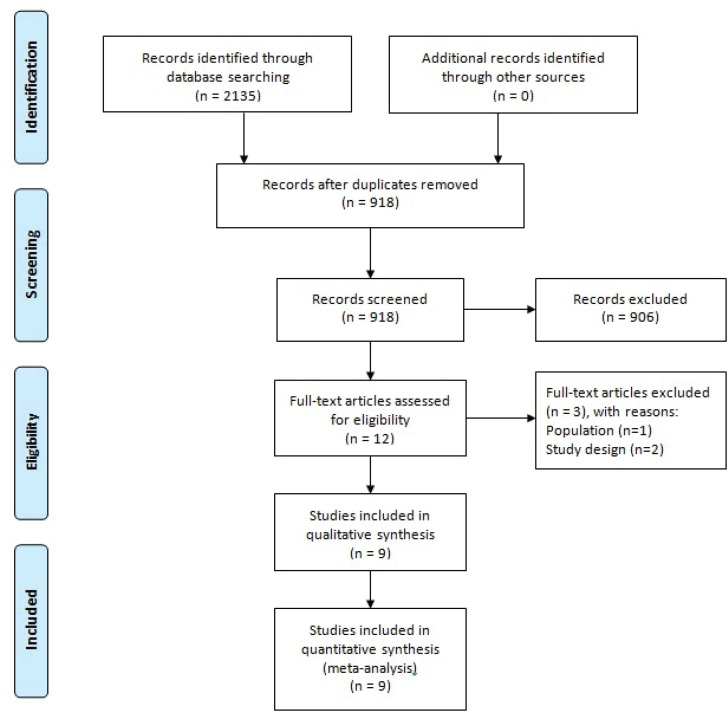
Prisma flow diagram of studies selection

Initial database searches retrieved 2135 citations, of which 918 citations remained after duplicate removal (Figure 1). The screening of titles and abstracts led to the identification of 12 potentially relevant articles. Forward and backward citation tracking did not result in the identification of additional relevant articles. Three of the 12 articles were excluded due to irrelevant population and design, leaving nine articles included in the analysis.

### Description of included studies

The characteristics of the included studies^14-18,20-22^ are presented in Table 1. All nine studies were two-arm (ECV attempted/not attempted) trials (two RCTs^21,22^ and seven NRSs^14-20^). The studies included 184704 pregnancies and were performed in Australia, Canada, Denmark, the UK, and the US. Various techniques and maternal positions were used for ECV. The most commonly used techniques were the ‘forward roll’ and ‘backflip’^16-20,22^, and the most common maternal position was left lateral tilt^16-22^. Ultrasound was performed before ECV to confirm breech presentation in several studies, and reactive non-stress cardiotocograms were commonly recorded^15-22^. The ECV technique was not described in two articles^14,15^. In eight studies, tocolysis was used^15-22^.

**Table 1:** Characteristics of the included studies

*Authors, Year, Country*	*Study design*	*Number in study group/Number in control group*	*Outcomes*
Balayla et al.^14^ 2014, Canada	Retrospective cohort study	6165/177158	Premature rupture of membranes (PROM), trial of labor, induction of labour, augmentation of labour, mode of delivery, precipitous labour, non-vertex presentation at birth, cord prolapse, antibiotic use, chorioamnionitis, meconium staining, abnormal tracing during labor, ventilation requirements, APGAR scores at 5 minutes, neonatal seizures, and Neonatal Intensive Care Unit admission.
Bewley et al.^15^ 1993, UK	Retrospective cohort study	51/39	Success of ECV, the failure to offer ECV when suitable, and undiagnosed breeches.
Brocks et al.^21^ 1984, Denmark	Randomized controlled study	31/34	Presentation at delivery, and method of delivery.
Dyson et al.^16^ 1986, USA	Prospective cohort study	158/40	Apgar score <7 at 1 and 5 minutes, nuchal chord, congenital anomaly, intrapartum vertex presentation, caesarean delivery, maternal hospital stay, and infant hospital stay.
Goh et al.^17^ 1993, Australia	Retrospective cohort study	32/40	External cephalic version success rate, and caesarean section rate.
Healey et al.^18^ 1997, UK	Retrospective cohort study	89/95	External cephalic version success rate, breech presentation rate (suitable for external cephalic version) at delivery, and delivery mode rate for breeches.
Nassar et al.^19^ 2006, Australia	Retrospective cohort study	399/161	Presentation at delivery, pre-labour rupture of membranes, antepartum haemorrhage, onset of labor within 24 hours, uterine rupture, placental abruption, cord prolapse, nuchal cord, and antepartum fetal death.
Stine et al.^20^ 1985, USA	Prospective cohort study	141/23	Presentations at delivery, caesarean section rate, indications for surgery, outcome for fetus; meconium, abnormal cord position, fetal distress, Apgar score <7 at 1 and 5 minutes.
Van Dorsten et al.^22^ 1981, USA	Randomized controlled study	25/23	Presentation at delivery, CS rate, Apgar score <7 at 1 and 5 minutes, enrolmentdelivery interval, birthweight, meconium during labour or at delivery.

All articles contained information about CS performance, the failure to achieve vaginal cephalic birth, non-cephalic presentation at birth, and vaginal breech birth. Some outcomes were not reported consistently, but data on them could be derived from summaries of findings (e.g. data on vaginal cephalic birth were acquired by combining data on vaginal breech birth and non-cephalic presentation at birth). Most authors^14-16,18-20^ did not report on all study outcomes. Neonatal outcomes were reported according to presentation, rather than allocation, in three articles^16,19,20^; these data were not included in the analyses.

### Risk of bias summary for included studies

The overall risk of bias was unclear for the two RCTs^21,22^ (Table 2). For these trials, the risk of selection bias was also unclear because the authors did not describe the method used to generate or conceal allocation sequences. The two studies were deemed to have a low risk of performance bias and a high risk of detection bias, as the authors did not explain the methods used to address blinding of the outcome assessors to intervention allocation. The risk of bias for outcomes was low in both studies. The risk of selective reporting was unclear because the authors did not examine the possibility of outcome reporting or what was found. Thus, insufficient information was available to assess the risk of reporting bias.

**Table 2:** Assessment of risk of bias in studies on obstetrical and neonatal outcomes following external cephalic version (Cochrane's risk of bias for randomized controlled studies)

*Authors, Year*	*Risk of bias wit hin each domain*							
	Random sequence generation (selection bias)	Allocation concealment (selection bias)	Blinding of participant and personnel (performance bias)	Blinding of outcome assessment (detection bias)	Incomplete outcome data (attrition bias)	Selective reporting (reporting bias)	Other sources of bias	Overall risk of bias across studies
Brocks et al.^21^ 1984	Unclear	Unclear	Low	High	Low	Unclear	Unclear	Unclear
Van Dorsten et al.^21^ 1981	Low	Unclear	Low	High	Low	Unclear	Unclear	Unclear

Judgement: Bias is assessed as a judgment (high, low, or unclear) for individual elements from five domains (selection, performance, attrition, reporting, and other). Assessment of risk of bias: Low risk of bias – low risk of bias for all key domains; Unclear risk of bias – unclear risk of bias for one or more key domains; High risk of bias – high risk of bias for one or more key domains.

The overall risk of bias was serious for the seven NRSs^14-18,20^ (Table 3). Three studies^14,16,19^ had low risks of confounding and the remaining four studies had serious risks of confounding because the authors did not describe whether they controlled for confounders. All seven NRSs were deemed to be at moderate risk of selection bias because the participants were not randomized. The risk of bias in the classification of interventions was low for five studies^16-20^ and serious for the remaining two studies^14,15^ because the ECV interventions were not described. All studies had low risks of bias due to deviations from the intended studies and to missing data, and serious risks of bias due to the lack of blinding of outcome assessors. All NRSs were deemed to be at moderate risk of reporting bias because the authors did not report pre-registered protocols or statistical analysis plans.

**Table 3:** The risk of bias in non-randomized studies of interventions (ROBINS-I)

*Authors, Year, Country*	*Risk of bias within each domain*							
	Confounding	Participant selection	Intervention classification	Departure from intended interventions	Missing data	Measurement of outcomes	Selection of reported results	Overall risk of bias
Balayla et al.^14^ 2014, Canada	Low	Low	Serious	Low	Low	Serious	Moderate	Serious
Bewley et al.^15^ 1993, UK	Serious	Low	Serious	Moderate	Low	Serious	Moderate	Serious
Dyson et al.^16^ 1986, USA	Low	Low	Low	Low	Low	Serious	Moderate	Serious
Goh et al.^17^ 1993, Australia	Serious	Low	Low	Low	Low	Serious	Moderate	Serious
Healey et al.^18^ 1997, UK	Serious	Low	Low	Moderate	Low	Serious	Moderate	Serious
Nassar et al.^19^ 2006, Australia	Low	Low	Low	Low	Low	Serious	Moderate	Serious
Stine et al.^20^	Serious	Low	Low	Low	Low	Serious	Moderate	Serious

Judgement: Low risk of bias (comparable to a well performed randomized controlled trial: RCT); Serious risk of bias (important problems); Moderate risk of bias (sound, but not comparable to a well performed RCT); Critical risk of bias (too problematic to provide useful evidence), no information.

### Effects of external cephalic version for breech presentation on outcomes of interest

#### Primary outcomes: failure to achieve vaginal cephalic birth and caesarean section

**Figure 2:**
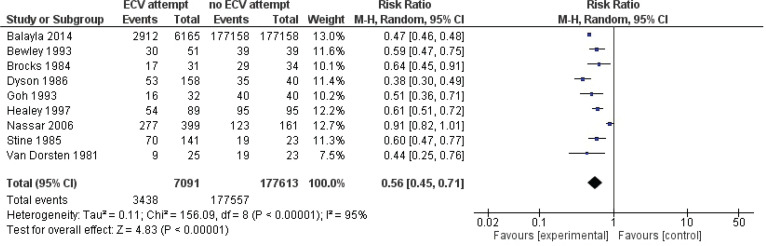
Vaginal cephalic birth not achieved (caesarean section + breech vaginal birth)

**Figure 3:**
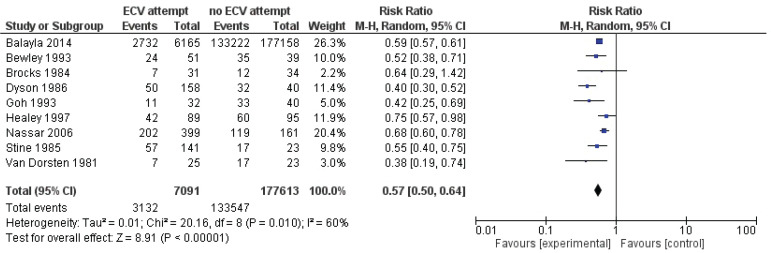
Caesarean section

Pooled data from all included studies showed that attempted ECV was associated with significant reductions in the failure to achieve vaginal cephalic birth (nine studies: RR=0.55; 95% CI: 0.44–0.66) (Figure 2) and the performance of CS (nine studies: RR=0.57; 95% CI: 0.50–0.64) (Figure 3). Conversion of the data to absolute numbers suggested that attempted ECV, relative to no ECV, would probably result in 340–530 fewer failures to achieve vaginal cephalic birth and 271–376 fewer cesarean sections per 1000 cases of labor (Table 4).

**Table 4:** Anticipated absolute effects for ECV-attempt versus no ECV-attempt

*Outcomes*	Risk with no ECV-attempt n*	Risk with ECV-attempt n* (95% CI)	*Relative effect RR (95% CI)*	*Participants (Number of studies)*
Vaginal cephalic birth not achieved (CS + breech vaginal birth)	1000	550 (470–660)	0.55 (0.47–0.66)	184704 (9)
Apgar score <7 at 5 min	19	24 (21–29)	1.29 (1.10–1.52)	184129 (4)
Caesarean section	752	429 (376–481)	0.57 (0.50–0.64)	184704 (9)
Non-cephalic presentation	1000	450 (290–680)	0.45 (0.29–0.68)	184704 (9)

* Study population per 1000 cases of labour. RR: relative risk.

#### Secondary outcomes: perinatal and maternal morbidity and mortality

**Figure 4:**
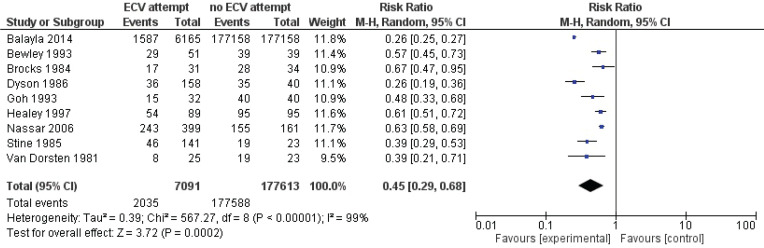
Non-cephalic presentation

**Figure 5:**
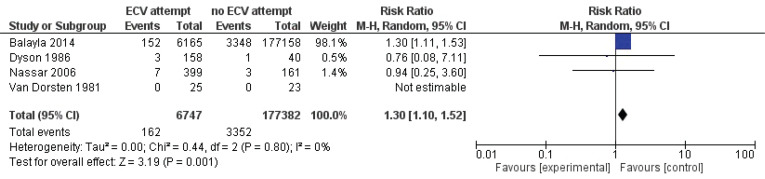
Apgar score at 5 min

Pooled data from all included studies showed that attempted ECV was associated with a significant reduction in noncephalic presentation at birth (three studies: RR=0.45; 95% CI: 0.29–0.68) (Figure 4) and an increase in low Apgar scores at 5 minutes (four studies: RR=1.29; 95% CI: 1.10–1.52) (Figure 5). Conversion of the data to absolute number suggested that attempted ECV, relative to no ECV, would probably result in 320–710 fewer non-cephalic presentations at birth and 2–10 more low Apgar scores at 5 minutes per 1000 cases of labor (Table 4). Attempted ECV had no effect on the incidence of vaginal breech birth (nine studies: RR=0.55; 95% CI: 0.24–1.24), low Apgar scores at 1 minute (nine studies: RR=0.85; 95% CI: 0.61–1.17), perinatal death (one study: RR=0.06; 95% CI: 0.00–1.11), or neonatal admission (two studies: RR=1.06; 95% Cl: 0.96–1.18).

## DISCUSSION

The pooled effect estimates from the nine studies included in this review show that attempted ECV at or near term reduces the risks of CS, non-cephalic presentation at term, and failure to achieve vaginal cephalic birth. Compared with no ECV, attempted ECV was also associated with a slightly increased risk of a low Apgar score at 5 minutes. It had no significant effect on the incidence of vaginal breech birth, low Apgar scores at 1 minute, perinatal death, or neonatal admission.

The findings of this review are in line with those of a 2015 Cochrane Review of eight RCTs comparing the effects of ECV and no ECV at term for breech presentation^7^, which revealed associations of ECV at term with significant reductions in non-cephalic presentation at birth, failure to achieve vaginal cephalic birth, and CS performance. The authors of that review, however, found no significant effect of attempted ECV on the incidence of low Apgar scores at 5 minutes or other measures of perinatal morbidity, and concluded that evidence from randomized trials was insufficient for the assessment of complications of ECV at term. Large observational studies suggest that such complications are rare^5^. The discrepancy in results between reviews might be explained by differences in design and populations. In the 2015 Cochrane Review^7^, Apgar scores <7 at 5 minutes were reported in five RCTs involving 428 infants. Women for whom ECV was attempted in those studies may have had fewer birth events resulting in such scores than did the women in the studies included in the present review. Our data suggest that slightly more birth events with subsequent low Apgar scores at 5 minutes occurred in the attempted ECV group than in the no ECV group (24 vs 19 per 1000). This finding is not surprising, as successful ECV prolongs pregnancy by avoiding elective CS and enabling labor, accompanied by small, but established, risks of morbidity and mortality^23^.

### Strengths and limitations

Applicability was of little concern for all included studies, implying that the evidence is relevant to current practice. The focus in this review on studies conducted in high-resource countries means that the findings may not be relevant for different, less well-resourced contexts. The recent trend of routine CS performance for persistent breech presentation may mean that the effect of attempted ECV on CS rates is greater than revealed in this review. All of the included studies had design limitations; most participants were aware of the intervention, which may have affected decisions about care during later pregnancy and birth. The healthcare staff also were aware of the intervention, which may have affected other aspects of care and decision-making during birth, impacting outcomes such as CS performance.

## CONCLUSIONS

Policy makers should review national and local guidelines on breech pregnancies to consider whether to offer attempted ECV at or near term to women with no contraindication. In this way, the risk of CS could be reduced and the strain on overburdened healthcare systems could be alleviated. The evidence examined in this review is limited by the scarcity of high-quality research and the presence of risks of bias.
