# Supplementary material for: Effects of external cephalic version for breech presentation at or near term in high-resource settings: A systematic review of randomized and non-randomized studies
Source: Eur J Midwifery. 2020 Nov 20;4:44. doi: 10.18332/ejm/128364 (PMC7839085; doi:10.18332/ejm/128364)
Supplement: Supplementary file 1 [file EJM-4-44-s1.pdf]

External cephalic version for breech presentation – search strategy

Electronic databases (Medline, Embase, CINAHL, Cochrane Library, MIDIRS, and SweMED+) were searched to identify eligible studies from the earliest year possible through April 2019, published in English or a Scandinavian language.

Database: Ovid MEDLINE(R) ALL

1 Breech Presentation/
2 breech.tw,kf.
3 1 or 2
4 Version, Fetal/ (793)
5 (external adj2 version*).tw,kf.
6 cephalic version*.tw,kf.
7 or/4-6
8 3 and 7
9 limit 8 to (danish or english or norwegian or swedish)

Database: EMBASE

1 breech presentation/
2 breech.tw,kw.
3 1 or 2
4 external version/ or external cephalic version/
5 (external adj2 version*).tw,kw.
6 cephalic version*.tw,kw.
7 or/4-6
8 3 and 7
9 limit 8 to (books or chapter or conference abstract or letter)
10 8 not 9
11 limit 10 to (danish or english or norwegian or swedish)

Database: Maternity & Infant Care Database (MIDIRS)

1 breech.mp.
2 External cephalic version.de.
3 (external adj2 version*).mp.
4 cephalic version.mp.
5 2 or 3 or 4
6 1 and 5

Database: Cochrane Library

#1 MeSH descriptor: [Breech Presentation] this term only
#2 (breech):ti,ab,kw
#3 #1 or #2
#4 MeSH descriptor: [Version, Fetal] this term only
#5 ((external NEAR/2 version*)):ti,ab,kw
#6 ("cephalic version"):ti,ab,kw
#7 #4 or #5 or #6
#8 #3 AND #7

Database: CINAHL

S1 MH "Breech Presentation"

S2 TX breech
S3 S1 OR S2
S4 MH "Version, Fetal"
S5 TX external N1 version* OR TX ("cephalic version" OR "cephalic versions")
S6 S4 OR S5
S7 S3 AND S6
Limiters - Language: Danish, English, Norwegian, Swedish

Database: Svemed+
Søkestrategi:
exp:"Version, Fetal"

PRISMA 2009 Flow Diagram

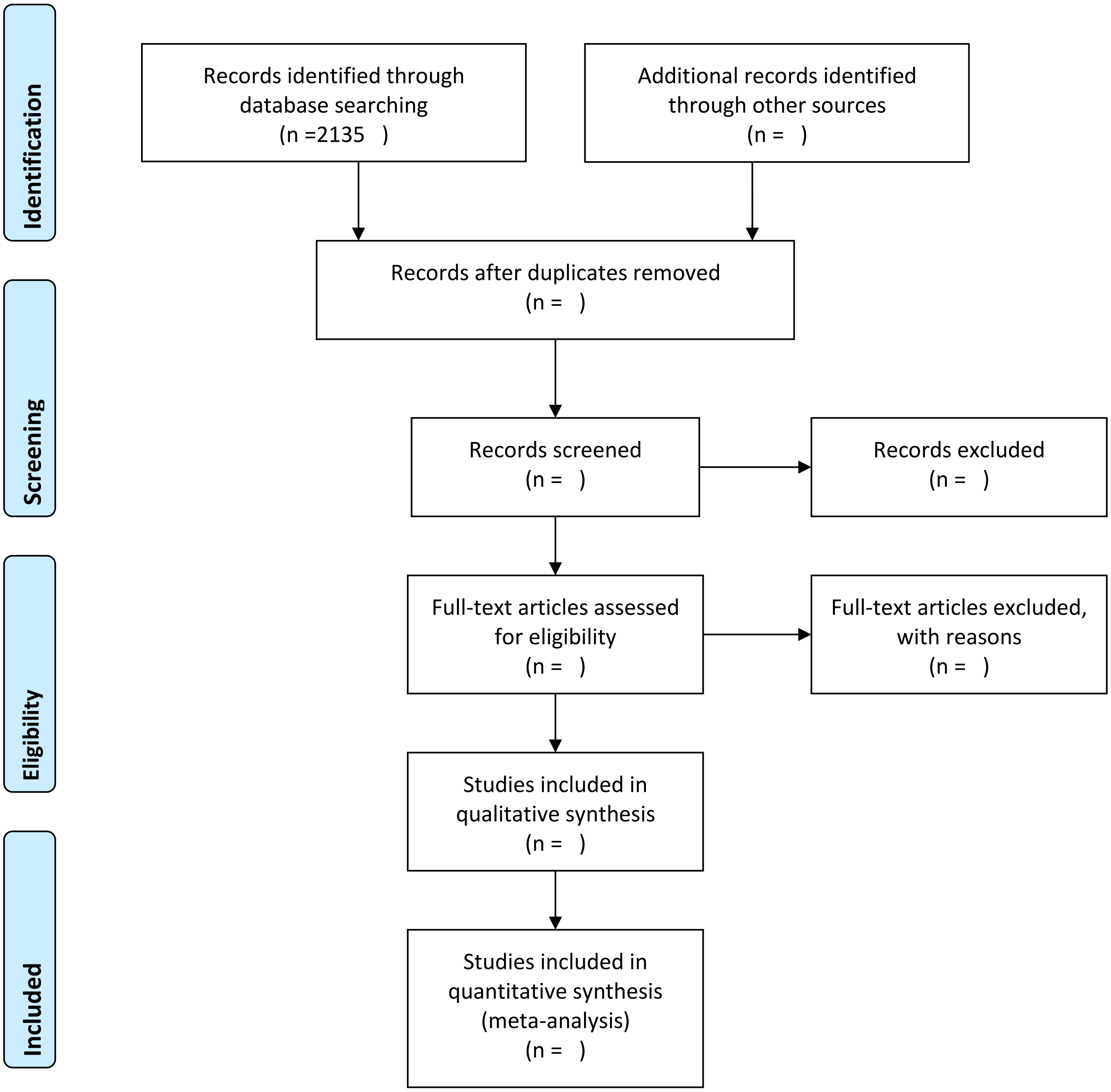

From:
Moher D, Liberati A, Tetzlaff J, Altman DG; PRISMA Group. Preferred reporting items for systematic reviews and meta-analyses: the PRISMA statement. PLoS Med. 2009 Jul 21;6(7):e1000097. doi: 10.1371/journal.pmed.1000097

For more information, visit www.prisma-statement.org.

© 2020 Devold Pay A. S. et al.

UiO : Universitetsbiblioteket
Medisinsk bibliotek
